# Epidemiological, clinical features and susceptibility pattern of shigellosis in the buea health district, Cameroon

**DOI:** 10.1186/1756-0500-5-54

**Published:** 2012-01-21

**Authors:** Anna L Njunda, Jules CN Assob, Dickson S Nsagha, Henri LF Kamga, Maghah P Awafong, Elroy P Weledji

**Affiliations:** 1 Department of Medical Laboratory Sciences, Faculty of Health Sciences, University of Buea, P.O.Box 63, Buea, Cameroon; 2 Medicine Programme, Department of Biomedical Sciences, Faculty of Health Sciences Laboratory, University of Buea, P.O.Box 63, Buea, Cameroon; 3 Department of Public Health and Hygiene, Faculty of Health Sciences, University of Buea, P.O.Box 63, Buea, Cameroon; 4 Department of Clinical Sciences, Faculty of Health Sciences, University of Buea, P.O.Box 63, Buea, Cameroon

**Keywords:** Shigellosis, Epidemiology, Clinical features, Antibiotics, Susceptibility pattern, Cameroon

## Abstract

**Background:**

Shigellosis is an acute invasive enteric infection caused by bacteria belonging to the genus *Shigella*; it is clinically manifested by bloody diarrhoea. Shigellosis is endemic in many developing countries including Cameroon and also occurs in epidemics causing considerable morbidity and mortality. This study evaluated the epidemiological and clinical features of *Shigella *and the resistance pattern of isolates to commonly used antibiotics in the Buea Health District in Cameroon, from April to August, 2010.

**Results:**

Of the 223 stool samples cultured, 10 (4.5%) yielded *Shigella *species. Isolation rate was observed to be more in children below 15 years (7.89%), and also higher in rural areas (6.35%). All 10 isolates showed resistance to at least two antibiotics and 9 (90%) were multi-drug resistant. The highest resistance rates were encountered with cotrimoxazole (90%) and amoxicillin (80%). Least resistance was observed with azithromycin (10%).

**Conclusion:**

Shigellosis is more prevalent in children below 15 years in the Buea District. There is a high level of resistance to most of the antibiotics used for the treatment of shigellosis including extended-spectrum beta-lactamases (ESBLs) as well as evidence of resistance to quinolones. Azithromycin was found to be the drug of choice for shigellosis in this setting.

## Background

Shigellosis (bacillary dysentery) is a global health problem, especially in developing countries, where substandard hygienic conditions and unsafe water supplies prevail [1]. Among the bacterial causes of dysentery, *Shigella *species continue to be the most important, with a high infectivity rate and the development of antimicrobial resistance. Shigellosis is said to be a self-limiting disease, although antimicrobial therapy is recommended [2]. Since 1940, when resistance of *Shigella *species to sulfonamide was first recognized in Japan [3], resistance of *Shigella *species have become progressively recorded to most widely-used antimicrobial agents [4]. Multiple-drug resistance transmitted plasmids among *Shigella *species have been reported in many countries [5]. So far, limited reports exist on the occurrence and antibiotic resistance patterns of *Shigella *species in Buea in particular and in Cameroon in general. The purpose of the present study was to determine the epidemiologic profiles of *Shigella *and their antimicrobial resistance patterns in the Buea Health District, in the South West Region of Cameroon.

## Results and discussion

Of the 223 stool specimens cultured for *Shigella *species, 10 were tested positive with a prevalence of 4.5%. Findings from other authors [6-8] indicate slightly higher prevalence rates of 8.0%, 7.4% and 7.3% respectively, than what is obtained in this study.

The majority of *Shigella *isolates were from the paediatric population (children < 15 years) with an infection rate of 7.89% while no infection was recorded with individuals aged > 45 years. In line with our findings, a higher prevalence of infection in children was observed in a previous work [9], in which 70% of all infections occurred in children below 15 years. It was also noted that children of this age group accounted for more than a third of all *Shigella *positive patients [8]. Scientific findings have revealed that type-specific immunity develops after repeated exposures during childhood [10]. Diarrhoea account for about 16% of all causes of mortality in children [11], because they are at an immature age, and they are in frequent exposure to contaminated environment due to play-related activities and are not being drilled on the importance of hand-washing after defecation and before eating.

Considering the source of water of these participants, it was noted that most participants 181 (81.17%) used pipe-borne water, while 38(17.04%) used springs and 4(1.79%) fetched from the river. Of the 10 isolates, 7(70%) occurred in patients whose drinking water was obtained from springs and 3 (30%) of patients who consume pipe-borne water with no significance (*p *> 0.05). Abdominal pain, fever and diarrhoea were the most common clinical symptoms. Out of the 60 (26.91%) patients that presented with diarrhoea and 9 (4.0%) with dysentery, 2(3.33%) and 2 (22.22%) respectively, yielded *Shigella *species. There was a significant difference between dysentery and infection (*p *< 0.05) while it was not the case with the other clinical conditions (*p *> 0.05). Of the ten positive isolates, 3(30%) were from formed and semi formed stool samples each while 2(20%) were from mucous and bloody mucous stool, each. Analyses of disease occurrence for shigellosis have faced difficulties, diagnosis especially at the clinical levels, are often missed because many cases are asymptomatic or present with atypical features such as watery diarrhoea and fever. Bloody diarrhoea has been the major clinical presentation associated to shigellosis [12]; nonetheless there exists other presentations which range from asymptomatic carriage, to mild watery diarrhoea, to overt dysentery characterized by frequent, but small volume, loose stools, consisting largely of blood and mucus [13]. It was observed in this study that although bloody diarrhoeas accounted for 22.22% of the infections; watery (11.76%), formed (2.4%) as well as semi-formed (5.56%) stools also yielded Shigella. Isolation of Shigella from formed and semi-formed stools is an indication that the patients were in the period of convalescence though still harboring the germ. Responses to questions administered to medical doctors revealed that, in the treatment of bloody diarrhoeas, stool cultures and antibiotic susceptibility testing were hardly ever requested due to lack of appropriate facilities in these treatment centers thus favoring the tendency of prescribing antibiotics on the basis of clinical diagnosis alone. Implying that diagnosis on the bases of clinical symptoms alone could be misleading. The misuse of antibiotics is considered the most important factor promoting the emergence, selection and dissemination of antimicrobial resistance observed with many bacteria [14,15]. This study also determined the sensitivity and resistance patterns of the isolates to various antibiotics commonly used for the treatment of shigellosis. From the results of the antibiogram, azithromycin (macrolide) was the most sensitive drug, with a sensitivity rate of 80% seconded by ofloxacin and ceftriaxone both of them recording sensitivity rates70% (Table 1). Though the sensitivity of azithromycin has not yet been recorded in previous studies, it was proved to be potent in this study, as an antimicrobial agent for use in management of shigellosis. The important sensitivity rates recorded by these drugs are probably due to the fact that they are less abused. Resistance to cotrimoxazole, one of the drugs used in the treatment of shigellosis has been reported in many studies. Belay et al. [16] demonstrated a 55% resistance rate of Shigella strains to cotrimoxazole while Khan-Mohammed et al. [17] recorded only 2.7% resistance rate to this drug in Trinidad. The present work showed a 90% resistance rate of the isolates to cotrimoxazole which is in agreement with observations from Yaoundé, Cameroon, Iran and Ghana [7,16] where resistance rates of 87.8%, 92% and 100% respectively, were reported. Unfortunately, the drug is still commonly prescribed and used in automedication by the community for diarrhoeal diseases and other infections due to its availability in local and pro-pharmacies at a very low cost. Amoxycillin and ampicillin were the next on the chart of resistant antibiotics, with resistance rates of 80% and 70% respectively (Table 1). Looking at Table 2, five Shigella isolates developed cross-resistance to these two antibiotics whereas 3 (against amoxicillin) and 2 (against ampicillin) were solely resistant to each of the amino-penicillins. They are beta-lactam penicillin antibiotic with bactericidal activity binding to and inactivating the penicillin-binding proteins (PBP) located on the inner membrane of the bacterial cell wall which interferes with the cross-linkage of peptidoglycan chains necessary for bacterial cell wall strength and rigidity [18]. The difference in resistance patterns of these 2 antibiotics may be due to the difference in administration routes. Ampicillin is administered frequently through parenteral routes whereas amoxicillin is orally administered. The latter is therefore more prone to abuse related to automedication and over prescription. Although they share in common the same resistance mechanism, some species are able to induce resistance to beta-lactams as a result of changes in porins in the outer membrane. Such changes decrease or eliminate the flow of small hydrophilic molecules like beta-lactams [18]. Similarly high rates of resistance to the above antibiotics of 88.5% and 78.5% respectively, were reported in Iran [19]. Wilson et al. [20] observed a resistance rate of 39.7% by Shigella isolates to chloramphenicol comparable with 40% resistance rate obtained in this study. Occurrence of Shigella species with multi drug resistance to 4 and 7 antibiotics (Table 2) including members of beta lactams (ampicilin and amoxicillin) and cephalosporins (Ceftriaxone) is precursor of the presence in the Buea Health District of extended-spectrum beta-lactamases (ESBLs) [21]. ESBLs are enzymes capable of hydrolysing penicillins, broad-spectrum cephalosporins and monobactams, and are generally derived from TEM and SHV type 3 or 4 enzymes. ESBL-producing enterobacteriaceae have been responsible for numerous outbreaks of infection throughout the world and pose challenging infection control issues. Antibacterial choice is often complicated by multi-resistance and often leads to therapeutic failure of empirical therapy; therefore knowledge of the local prevalence of pathogens and their antimicrobial sensitivity patterns is essential for clinicians in their routine work [15,21]. Risk factors associated with the spread of these dangerous strains is due to indiscriminate prescription of 3rd generation cephalorins (ceftriaxone) and risk factors such as living in poor hygienic conditions like in Buea health district [22]. Penicillins and chloramphenicol are broad-spectrum antibiotics commercially available and affordable to the public for treatment of enteric, urinary tract and ocular infections; thus they are often purchased on the basis of self-prescription and administered at sub-therapeutic dosages, accounting for the high rates of resistance observed. Nalidixic acid and ofloxacin showed resistance rate of 60% and 20% respectively (Table 1), implying that quinolone resistance is spreading in Cameroon [15]. In a previous study a resistance rate of enterobacteriaceae of 25.7% against quinolones has been observed. Although the quinolone resistance mechanism described most frequently, both in Shigella spp. and in other microorganisms, involves the presence of mutations in the quinolone target site; nalidixic acid resistance is related mainly to the presence of a single amino-acid substitution at either position 83 or position 87 of GyrA, while resistance to ciprofloxacin is related to the presence of at least one additional substitution in GyrA or ParC [23]; this difference may also be due to the presence of efflux pumps in Shigella spp. that are able to confer quinolone resistance in the absence of GyrA substitution. The widespread use of these agents might account for the high percentage of quinolone-resistant isolates in the study area[24] as compared with findings from Mache [25] who reported a resistance rate of 6.5%; and Andrea et al. [17] who recorded 40%. This shows a constant increase in resistance rates as the years go by. The same trend applies to gentamicin; Gizachew et al. [8] in a comparative study of the resistance rate of Shigella species to commonly used antibiotics in different years in Ethiopia observed a fourfold increase in resistance to gentamicin; from 2% as stated by Assefa et al. [26] to 7.9% by Gizachew et al. [8]. They attributed this change to an increase in the use of the drug over the past 5 years. The resistance rate obtained with gentamicin in the present study (40%) (Table 1) was higher than those reported in the above studies. Multiple-drug resistance to as many as seven antibiotics was observed here (Table 2) and this is similar to Belay et al. [16] and Wilson et al. [20]'s findings where resistance to six and seven antibiotics were found, among which ampicillin, cotrimoxazole and gentamicin, showed the highest occurring resistance pattern. Murray et al. [27] contended that resistance is encountered more with those antibiotics misused or used frequently for therapeutic and/or prophylactic purposes. Cotrimoxazole, ampicillin and amoxycilin are easily obtained outside of recognized treatment centers and taken without medical supervision at sublethal dosages and during an insufficient length of time. Buea, being a cosmopolitan town and owing to the fact that a significant portion of the population is made up of university students; there is a frequent movement in and out of the town. This mobile population provides opportunities for the rapid spread of multi-resistant organisms in regions where unrestricted antibiotic use is common, scarcity of potable water combined with many factors favouring live in poor hygienic conditions. Unless the unrestricted use of these antibiotics is stopped in this area, the time that these antibiotics become ineffective in the treatment of bacillary dysentery and other infectious agents is not far.

**Table 1:** In vitro antimicrobial susceptibility pattern of isolates

ANTIBIOTIC CLASS	ANTIBIOTIC TESTED	*NUMBER (Percentage)*		
		
		Sensitive	Intermediate	Resistant
**Quinolone**	Nalidixic acid	2 (20)	2 (20)	6 (60)

**Fluoroquinolones**	Ciprofloxacin	4(40)	6 (60)	0 (0)

	Ofloxacin	7 (70)	1 (10)	2 (20)

**Macrolide**	Azithromycin	8 (80)	1 (10)	1 (10)

**Cephalosporin**	Ceftriaxone	7(70)	1(10)	2(20)

**Aminoglycoside**	Gentamicin	4 (40)	2 (20)	4 (40)

**Sulfonamide and trimethoprime**	Cotrimoxazole	1 (10)	0 (0)	9 (90)

**Penicillins**	Amoxycillin	2 (20)	0 (0)	8 (80)
	Ampicillin	2 (20)	1 (10)	7 (70)

**Chloramphenicols**	Chloramphenicol	4 (40)	2 (20)	4(40)

*χ*^2 ^= 3.93, df = 4, *p *= 0.42

**Table 2:** Drug resistance patterns of *Shigella *isolates

*Resistance patterns *	*Antibiotics*	*Number and percentage of resistant isolates n = 10)*
2 antibiotics	AMX, A	1 (10)

3 antibiotics	AMX, NA, SXT	1 (10)

4 antibiotics	A, C, NA, SXT	1 (10)

	A, CN, CRO, SXT	1 (10)

	AMX, A, CN, SXT	2 (20)

5 antibiotics	AMX, A, C, NA, SXT	1 (10)

	AMX, A, NA, OFX, SXT	1 (10)
	AMX, A, C, NA, SXT	1 (10)

7 antibiotics	AMX, C, CN, CRO, OFX, NA, SXT	1 (10)

AMX: Amoxycillin; A: Ampicillin; AZM: Azithromycin; C: Chloramphenicol; CIP: Ciprofloxacin; CN: Gentamicin; CRO: Ceftriaxone; OFX: Ofloxacin; NA: Nalidixic acid; SXT: Cotrimoxazole

This study which highlights the epidemiologic pattern of shigellosis in the Buea Health District however has a limitation including the inability to determine the species of *Shigella *or to serotype them. Information that could have given an idea of the severity of the species involved. However according to WHO [13], reporting any isolate of *Shigella *species is a major step in disease surveillance and helps in taking actions that lessen the risk of serious complications, spreading and death in the population.

## Conclusions

This work has revealed that the prevalence rate of shigellosis in the Buea Health District in Cameroon stands at 4.5%. The majority of *Shigella *isolates are from the paediatric population (children < 15 years) with an infection rate of 7.89%. This condition is linked to the poor hygienic conditions prevailing in the area. The antibiotic susceptibility testing has revealed that these Shigella spp are multiresistant to currently used antibiotics including extended-spectrum beta-lactamses (ESBLs) as well as increasing evidence of resistance to quinolones. Azithromycin was found to be the drug of choice for shigellosis in this setting.

## Methods

A total of 223 individuals comprising of 90 males and 133 females, aged 1 month to 72 years old were recruited in the study. The participants were patients referred to the laboratory for stool analysis from the in-patient and out-patient departments of the Regional Hospital Annex, Buea and the Kahwa Sumbele Medical Centre Bomaka, all within the Buea Health District. Their informed consent was souk and obtained and an ethical clearance and authorization to collect specimen and data for research was obtained from the South West Regional Delegation of Public Health Ref No. R11/MPH/SWR/RDPH/FP/5489/97 of the 06/04/2010. An estimate of the required number of participants was obtained using a formula for estimating sample size for proportions. The prevalence used in this formula was derived from a previous work [6]. Isolation, morphological and biochemical identification of Shigella spp were done according to standard methods [28]. The confirmed isolates were subjected to antimicrobial susceptibility tests by disc diffusion method [29-31].

(For details of the methods used see Additional file 1).

## Competing interests

The authors declare that they have no competing interests.

## Authors' contributions

ALN as principal investigator designed and implemented the project and initiated the writing of the manuscript. JCNA assisted in designing the project and revised the manuscript. HLFK assisted in the *in vitro *antimicrobial testing and in revising the paper. DSN assisted in the identification of microbial strains, the statistical analysis of data and in revising the paper. MPA provided technical assistance during antimicrobial testing. EPW assisted during the consultation in making sure that patients enrolled in the study presented with signs and symptoms of diarrhoea, he read and approved the final version of the manuscript.

## Supplementary Material

Additional file 1 **Methods**. Click here for file
