# Supplementary material for: Epidemiological, clinical features and susceptibility pattern of shigellosis in the buea health district, Cameroon
Source: BMC Res Notes. 2012 Jan 21;5:54. doi: 10.1186/1756-0500-5-54 (PMC3285523; doi:10.1186/1756-0500-5-54)
Supplement: Additional file 1 — Methods. [file 1756-0500-5-54-S1.RTF]

Methods
Study area
Buea is the capital of the South-West Region of Cameroon with a population of 200,000 inhabitants. The population has doubled within the last ten years, influenced by the presence of the University of Buea that was created in 1993, and numerous professional, secondary and high schools. The town is located 15kms away from the seashore, and 60 kilometres away from Douala, the economic capital of Cameroon. Buea is comprised of a multi-ethnic population, dominated by the Bakweri people who are the historic owners of the land. It is a fertile volcanic area lying on the slope of Mount Cameroon, the second highest mountain in Africa. Inhabitants are principally engaged in agriculture as the main economic activity, conditioned by two seasons: the dry and wet seasons. Almost all the 254 ethnic groups found in Cameroon are represented in the area, attracted by the fertile volcanic soil and the Cameroon Development Corporation (CDC), a giant agricultural corporation. Hygiene and sanitation within the municipality has been largely compromised by the sudden increase in population, thus the generation of huge amounts of domestic waste. The town suffers permanently from water scarcity though with fairly regular but inadequate electricity supply. 
Study design
A cross sectional hospital-based study conducted to determine the prevalence of shigellosis and the antimicrobial resistance patterns. Consenting patients of all age groups for routine stool analysis without or without diarrhoea or dysentery in the participating health care centres were included in the study. DiarrheaDiarrhoea was defined as as three or more loose bowel movements during a period of 24 hours. Dysentery was defined as one or more loose bowel movements with visible blood.  Fever was defined as an axillary temperature ≥37.5°C.  An estimate of the required number of participants was obtained using the formula for estimating sample size for proportions. 
S= z2 (p (1-p)/e2) where S= sample size; z degree of confidence to consider in the results; p= an estimate of the proportion of people falling into the group of interest in the population.  Here a prevalence rate of 7.4% was considered obtained from a study on pathogenic microorganisms associated with childwoodchildhood diarrhoea in Low-and –Middle Income countries: a case study of YaoundeYaoundé, Cameroon [6]. 
P=0.074; e= the proportion of error accepted (5%or e=0.05). From this formula S = 105.3
Study population 
A total of 223 individuals comprising of 90 males and 133 females, aged 1 month to 72 years old were recruited in the study. The participants were patients referred to the laboratory for stool analysis from the in-patient and out-patient departments of the Regional Hospital Annex, Buea and the Kahwa Sumbele Medical Centre Bomaka, all within the Buea Health District. Their informed consent was souk and obtained and an ethical clearance and authorization to collect specimen and data for research was obtained from the South West Regional Delegation of Public Health Ref No. R11/MPH/SWR/RDPH/FP/5489/97 of the 06/04/2010.
Specimen collection
Fresh single stool specimens were collected in sterile disposable containers avoiding contamination with urine.  Specimens were labelled with unique code numbers and transported to the Faculty of Health Sciences laboratory, University of Buea in ice cold boxes within two hours of collection. Analysis was done on arrival without any delay. 
Bacteriological analysis
For optimal isolation of Shigella, three different culture media were used in this study; Eosin Methylene Blue agar (a differential medium), Hektoen enteric agar (a selective medium) and Salmonella and Shigella agar, (a selective medium). These media contain bile salts to inhibit the growth of other Gram-negative bacteria and pH indicators to differentiate lactose fermenters (Coliforms) from non-lactose fermenters such as Shigella. For formed stools, a small portion was emulsified in sterile physiologic saline and a drop transferred to Eosin Methylene Blue agar (Scharlau Chemie S.A. Barcelona, Spain), Hektoen enteric agar (Scharlau Chemie S.A. Barcelona, Spain) and Salmonella-Shigella agar (Plasmatec, Bridport, UK). A loopful of liquid stools was plated directly onto the media. The plates were incubated aerobically at 37°C overnight. Convex, colourless (non-lactose fermenter), flat colonies with rough edges, 2-3 mm in diameter on Eosin Methylene Blue agar and Salmonella and Shigella agar; and green colonies, 2-3 mm in diameter on Hektoen enteric agar were suggestive of Shigella species [28,29].
Morphological and biochemical Identification 
Following overnight incubation of primary isolation media at 37° C, suspect colonies were stabbed and streaked into tube slants of Kligler's Iron Agar (KIA) (Liofilchem S.A. Italy) and stabbed in Motility Indole Urease (MIU) (Liofilchem S.A. Italy) medium. After overnight incubation in these differential media, Shigella species produced an alkaline slant and an acid but with no bubbles of gas in KIA and growth observed along the line of stab in MIU with the surrounding medium remaining clear [28,297]. This gave a presumptive but not conclusive identification of Shigella species. 
Confirmation of isolates 
Slide agglutination test with polyvalent antiserum (Liofilchem S.A. Italy) reconstituted following the manufacturer's instructions, was used to confirm the isolates. Colonies from the slant portion of the KIA tubes were emulsified into physiologic saline to make suspensions at the two ends of a clean slide, for test and negative control. One drop of antiserum was placed in the test well and noting added to the Negative control well. The suspensions were mixed until the colonies were dissolved, then placed on a rotator for one minute. Agglutination in the test well confirmed the isolate as Shigella [287]. Confirmed isolates were stored on Nutrient agar (Fluka, Biochemika; Germany) slants.
Antibiotic susceptibility test 
The confirmed isolates were subjected to antimicrobial susceptibility tests by disc diffusion method. Mueller-Hinton agar (Plasmatec, Bridport, UK), poured to a uniform depth of about 4 mm, was used for susceptibility testing. Three colonies were scooped and transferred to a tube containing 4ml of sterile normal saline and shacked thoroughly using a vortex [308]. A sterile cotton swab was dipped into the suspension and used to streak over the dried surface of Mueller-Hinton agar. The antimicrobial discs were dispensed at equidistant apart onto the surface of the inoculated agar plate using a sterilized forceps. The plates were inverted and incubated at 37ºC overnight [319].
After 24 hours incubation, the diameter of the zones of complete inhibition (including the diameter of the discs) was measured in millimeters using a caliper and compared with the zone-size interpretative table and recorded as susceptible, intermediate or resistant to each drug tested [10]. A total of ten antibiotics (Liofilchem S.A. Italy); ciprofloxacin (CIP, 5ìg), ampicillin (A, 10ìg), amoxycillin (AMX, 10ìg), chloramphenicol (C, 30ìg), nalidixic acid (NA, 30ìg), ceftriaxone (CFT, 30ìg), azithromycin (AZM, 15ìg), cotrimoxazole (SxT, 25ìg), ofloxacin (OFX, 5ìg) and gentamicin (CN, 10ìg) were tested against the isolates.
Statistical analysis
Data from the questionnaires and from laboratory analysis were entered into Microsoft excel software. Statistical analysis was done using the Statistical Package for Social Sciences (SPSS) version 11.0 software. The Chi-square test was used for the analysis of categorical variables. P-values < 0.05 were considered significant.
